# Evaluation of a person‐centred care model for persons living with dementia in the hospital rehabilitation setting using the RE‐AIM framework

**DOI:** 10.1002/alz.092089

**Published:** 2025-01-09

**Authors:** Claire V Burley, Anna Williams, Genevieve Maiden, Patricia Reyes, Jacquelene Cook, Henry Brodaty, Lynnette Chenoweth

**Affiliations:** ^1^ University of New South Wales, Sydney, NSW Australia; ^2^ Western Sydney University, Sydney, NSW Australia; ^3^ South Eastern Sydney Local Health District, Sydney, NSW Australia

## Abstract

**Background:**

One in four persons living with dementia are admitted to hospital, presenting challenges to them, their carers and staff. Despite global evidence demonstrating the clinical and cost‐effectiveness of person‐centered care (PCC), it is not yet *business as usual* across healthcare settings. We used multi‐level stakeholder input to implement Kitwood’s PCC model into a sub‐acute setting.

**Methods:**

Between June 2021 to June 2023, the PCC model was implemented and evaluated using the Consolidated Framework for Implementation Research (CFIR) and quantitative and qualitative methods to address the Reach, Effectiveness, Adoption, Implementation, and Maintenance (RE‐AIM) framework. Key stakeholders and a strong governance structure including persons living with dementia, family caregivers, clinicians, nurses, hospital leaders, community partners, government officials and policy makers, informed all aspects of implementation and evaluation. Co‐primary outcome measures were delirium and agitation. Data were collected pre‐, post‐ and 6‐months following PCC education.

**Results:**

Reach: Ninety nursing, medical and allied health staff (93% of hospital staff) completed online and/or in‐person PCC education; eight senior staff leaders completed in‐person PCC education and took on the role of ‘PCC Champion’ to support staff in implementing PCC. Effectiveness: Significant improvements (all *p*<.05) were observed in outcomes for persons living with dementia: delirium (phi = 0.73), incidents/injuries (phi = 0.99), psychotropic medication use (phi = 0.09), and readmissions (phi = 0.25). No change was observed for agitation. Adoption: Staff knowledge of and confidence in delivering PCC significantly improved (both *p*<.005). A significantly higher number of positive (person‐centered) interactions was observed following education (p = 0.000, phi = 0.60), though there was also a high number of neutral interactions. Implementation: PCC education delivery could and was efficiently adapted as required to unplanned crises (i.e. COVID‐19 pandemic). Maintenance: Six‐month follow‐up data showed staff maintained improved PCC knowledge and confidence (both *p*<.005). However, only three of the eight PCC Champions remained. Suggested modifications to aspects of the model include adopting reflective feedback processes with staff, PCC education for non‐clinical staff, reducing online content to fewer modules, and adapting the education to suit specific cultural groups.

**Conclusions:**

The successful translation of a PCC education model into a sub‐acute rehabilitation setting during a global pandemic has implications for policy and practice.

